# Chlorophyll soft-sensor based on machine learning models for algal bloom predictions

**DOI:** 10.1038/s41598-022-17299-5

**Published:** 2022-08-08

**Authors:** Alberto Mozo, Jesús Morón-López, Stanislav Vakaruk, Ángel G. Pompa-Pernía, Ángel González-Prieto, Juan Antonio Pascual Aguilar, Sandra Gómez-Canaval, Juan Manuel Ortiz

**Affiliations:** 1 Universidad Politécnica de Madrid, Madrid, Spain; 2 European Regional Centre for Ecohydrology of the Polish Academy of Sciences, Lodz, Poland; 3 IMDEA Water Institute, Madrid, Spain; 4 Universidad Complutense de Madrid, Madrid, Spain; 5 Instituto de Ciencias Matemáticas (CSIC-UAM-UCM-UC3M), Madrid, Spain

**Keywords:** Environmental sciences, Environmental impact, Machine learning

## Abstract

Harmful algal blooms (HABs) are a growing concern to public health and aquatic ecosystems. Long-term water monitoring conducted by hand poses several limitations to the proper implementation of water safety plans. This work combines automatic high-frequency monitoring (AFHM) systems with machine learning (ML) techniques to build a data-driven chlorophyll-*a* (Chl-*a*) soft-sensor. Massive data for water temperature, pH, electrical conductivity (EC) and system battery were taken for three years at intervals of 15 min from two different areas of As Conchas freshwater reservoir (NW Spain). We designed a set of soft-sensors based on compact and energy efficient ML algorithms to infer Chl-*a* fluorescence by using low-cost input variables and to be deployed on buoys with limited battery and hardware resources. Input and output aggregations were applied in ML models to increase their inference performance. A component capable of triggering a 10 $\upmu$g/L Chl-*a* alert was also developed. The results showed that Chl-*a* soft-sensors could be a rapid and inexpensive tool to support manual sampling in water bodies at risk.

## Introduction

Human development has exerted an intense pressure on water bodies through habitat modification, water pollution, and overexploitation of natural resources. In this context, eutrophication is one of the major threats to the health of aquatic ecosystems^1^, and harmful algal blooms (HABs) are its most worrying consequences in inland waters^2,3^. The combined effect of rising temperatures and the excessive nutrients loads has induced algal proliferation in many lakes and reservoirs around the world^4–7^. In particular, toxic cyanobacteria have been especially pervasive in freshwater^8^. High cyanobacterial biomass on surface water represents a serious risk for ecosystem and human health, as they cause bad odors and tastes, turbid waters, dissolved oxygen (DO) depletion, and animal poisoning by producing a wide range of toxins^9,10^. This implies a major socio-economic impact on affected areas in terms of public health, fisheries, tourism, monitoring and management plans^11,12^, the solution to which is to equip stakeholders and policy makers with rapid and inexpensive tools to understand, predict and cope with HABs.

Long-term water monitoring is one of the most important elements in mitigating the risks associated with HABs, therefore the World Health Organization (WHO) has proposed an Alert Levels Framework to recognize critical scenarios and apply water safety plans^13^. According to the decision tree provided by the WHO, three threshold levels could be recognized based on cells quantification (or equivalents in chlorophyll-a [Chl-*a*] and biovolume), microscopic examination and toxins analyses. The monitoring and management sequence progress in vigilance level, alert level 1 and alert level 2, as cyanobacteria are observed, cells number increase, and toxins are detected. Therefore, the collection of water samples by hand and the subsequent laboratory analysis are imperative to classify the risk. However, this manual sampling is often limited by the variable dynamics of blooms in time and space. Many HABs are ephemeral (hours or days) and are dispersed in patches throughout water bodies^14^, which contrast with the usually low periodicity of sampling and their collection at fixed stations. Higher manual sampling frequency at multiple points is usually economically and logistically unfeasible in long-term monitoring. This, together with long waiting periods for laboratory analysis, may hamper the effectiveness and responsiveness of the proposed water safety plans.

New technologies and methods have been developed to support manual sampling, as they track the spatial-temporal distribution of HABs more efficiently^15^. One of the major breakthroughs for continuous and autonomous water monitoring are the automatic high frequency monitoring (AHFM) systems^16,17^. These systems allow to collect a massive water quality data by deploying wireless sensor networks on floating platforms or buoys, thus facilitating decision-making based on real-time data. Recent advances in sensing technologies have increased the measurement frequency and the number of variables being monitored in water^18^, many of them related with algal and cyanobacterial growth; e.g. photosynthetic pigments (i.e. chlorophylls, phycocyanins, phycoerythrins), turbidity, nutrients, pH, water temperature, DO, electrical conductivity (EC), etc. AHFM systems are also compatible with other monitoring approaches, such as satellite imagery and unmanned aerial vehicles (i.e. drones), which allow the monitoring area to be expanded but at a lower sampling frequency (^19–21^). However, monitoring based on AHFM systems could still be problematic due to the limitations of some of the most critical sensors, generating biases between different monitoring approaches^22^. For instance, fluorescence sensors (also called fluorometers or optical sensors) are widely used for the quantification of Chl-*a* and phycocyanin, as they provide an estimation of algal and cyanobacterial biomass. However, interferences caused by certain environmental factors, such as temperature and light^23,24^, which also vary according to manufacturer and cyanobacterial density^25^, may lead to inaccurate results. Another challenge to account is the selection of the adequate sampling point and depth. Several of the most pernicious cyanobacterial species are buoyant, and their position in the water column varies during the day^26^. Also, they can form scums and be accumulated in a thick surface layer or be pushed towards the shore area by winds and waves^27^. Thus, sensors placed on buoys anchored to fixed sampling points may be blinded due to the specific dynamics of HABs in such an environment; and hence, the interpolation of the data along the waterbody may not be representative^28–30^.

To reduce measurement uncertainty, stakeholders could consider increasing the number of sensors deployed in the area of interest. They might also contemplate placing fluorescence sensors at different depths or using vertical-profiling sensor platforms^31^. However, since fluorescence sensors are usually the most expensive ones, they will face financial and operational difficulties in implementing such a strategy, especially in large and heterogeneous water bodies. As an alternative to the inherent difficulties of fluorescence sensors, deploying AHFM systems composed by common low-cost sensors could be a solution to increase the sampling area. The correlation of certain physicochemical parameters with bloom-forming cyanobacteria is widely known, as is the case of temperature and pH^32,33^. Even variables such as EC, of great interest for inferring salinity, rainfall events and mixing of water column, have been associated with certain toxic species of cyanobacteria^34^. Although these sensors cannot substitute laboratory analysis by themselves, with the sustenance of advances in artificial intelligence and machine learning (ML), they can help to provide significant information for inferring Chl-*a* at those locations where the deployment of fluorescence sensors is not possible or not economically viable. Therefore, Chl-*a* can be estimated over large sampling areas in real time, leading to more accurate and timely manual sampling, and thus helping to trigger alerts on HABs outbreaks.

In recent years, software-based sensors (so-called soft-sensors) have become indispensable in many different areas and applications as an alternative to sensors implemented in the form of physical entities. In these applications, many variables that are monitored through physical sensors are difficult to measure or can only be measured sporadically due to the high cost of the sensors. Moreover, in some cases, the variables are measured with long delays due to the slowness of the hardware sensors or laboratory analysis, which leads to the impossibility of monitoring the process under study in real time. Broadly speaking, there are two types of soft-sensors^35–37^: (i) model-driven (or white-box), which have all the phenomenological knowledge about the process behavior; and (ii) data-driven (or black-box), which are based on historical information about the process. Data-driven soft-sensors are the most frequently implemented and are very useful to infer variables that cannot be measured automatically at all, or that can only be measured at high cost, sporadically, or with long delays (e.g., by laboratory analysis). To date, different examples of data-driven soft-sensors have been proposed to infer water quality variables, such as total reactive phosphorus, nitrogen as nitrate and nitrogen as ammonia^38^; *Escherichia coli *concentration^39^; chlorine^40^; chemical oxygen demand^41^, among others.

The use of inferential ML techniques in data-driven soft-sensors has become popular due to the efficiency improvements provided by the large amount of available data and new emerging techniques^42^, such as artificial neural networks^43,44^, tree-based models^45^, support vector machines^46,47^ and random forests^48,49^. When recent historical data is available, soft-sensors based on inferential data can be implemented straightforwardly using supervised ML regressors. The historical data is used to allow the ML regressors to learn to infer the high-cost/hard-to-measure variable using only the low-cost/easy-to-measure variables as input. The performance of ML regressors depends on the complexity of the model and the amount of historical data available. In general, there is a trade-off between model performance and complexity, with more complex models typically requiring larger amounts of ram memory and disks for storage, as well as higher power consumption and more powerful CPUs to run the model and produce real-time inferences. Therefore, when computational resources are limited (i.e, memory, CPU or battery constraints), it is common to sacrifice some performance for the sake of deploying the model on low-cost hardware.

The idea of applying ML regression models to infer Chl-*a* has been previously explored in oceans, rivers, lakes and reservoirs. Several studies have proposed to infer Chl-*a* by feeding models with different water quality variables collected in situ, at different measurement frequencies and time periods. For instance, Wei et al.^50^ studied correlations between Chl-*a * concentration and five physicochemical factors of water (i.e., salinity, water temperature, depth, DO and pH) by collecting 2100 measurements during an artificial upwelling process in the ocean. Soro et al.^51^ inferred Chl-*a* by collecting 150 measurements of rainfall, water discharges and water quality data (*i.e.* water temperature, pH, EC, DO, salinity, phosphate, nitrate and ammonia) for 2 years in three main rivers in West Africa. Likewise, Shin et al.^52^ used different ML models to predict Chl-*a* concentrations along the Nakdong River in Korea. They trained one-day-ahead Chl-*a* forecasting models considering several time lags and a dataset with daily measurements of water quality (i.e. water temperature, pH, EC, DO, and total organic carbon) and weather variables (i.e. air temperature, sunshine, rainfall, inflows, and outflows). Unfortunately, a coarse granularity of one day may not be sufficient to apply ML models to a real-time monitoring system, where significant changes in biological variables, such as Chl-*a*, must be detected as soon as they appear. In addition, there are other studies that include variables of high cost (e.g., optical turbidity sensor) or difficult to measure (e.g., phytoplankton abundance) as input variables for ML models^43,53–56^. Furthermore, several works proposed the prediction of Chl-*a* as a time series using past Chl-*a* values. In Cho et al.^57^ ANN and LSTM models using daily time series were proposed to predict Chl-*a* concentration at 1 and 4 days in advance. Yu et al.^58^ employed LSTM models with monthly time series that were additionally smoothed using wavelets. In Shamshirband et al.^59^, authors apply ensemble methods built on the basis of ANN models on a daily time series and also transform the data using wavelets as in^58^. The measurement frequency and time periods of these studies were diverse, as measurements ranged on a scale of minutes, days or months, during short (days) and long (years) time periods. Interestingly, Barzegar et al.^60^ used a hybrid CNN-LSTM deep learning model to infer Chl-*a* and DO in the Small Prespa Lake in Greece. They used a dataset composed of easily measurable water quality variables, such as pH, oxidation-reduction potential, water temperature, and EC, collected at 15-minute intervals but for one year. However, none of these studies have analyzed the behavior of their trained ML models during the different months of the year, either because of the low frequency of measurements or because the study periods were not longer than one year. This is of paramount importance to evaluate the accuracy of the designed soft-sensors at the most critical times, i.e., when the bloom begins or when it dies and toxic cyanobacterial cells release toxins. Moreover, neither of them have so far evaluated the effectiveness of trained ML models in triggering the HAB alert levels.

In sharp contrast, our study covers some of the above-mentioned shortcomings of previous works. We deployed two buoys equipped with multiparametric probes containing the same low-cost and fluorescence sensors in two different areas in the As Conchas reservoir in NW Spain. Massive data of water temperature, pH, EC and fluorescence of Chl-*a* were collected from surface water for 3 years at intervals of 15 minutes. In addition, the power from solar panels recharging the batteries of the sensors deployed was also monitored as a cheap estimate of daylight hours. Besides, given the above-mentioned uncertainties that fluorescence sensors for Chl-*a* may exhibit, the data collected by the basin organization in charge of reservoir management (Miño-Sil Hydrographic Confederation) was also taken into account to understand the Chl-*a* soft-sensor behavior. This basin organization usually performs manual sampling in different points of the waterbody, which varies from fortnightly to monthly depending on the time of year (data shown in Figure S1 in the supplementary material). According to their reports, the study site has long history of summer HABs dominated by toxic cyanobacteria, mainly of the genus *Microcystis*. Therefore, this study provides valuable information for stakeholders to implement a well-designed fieldwork and to achieve a reliable and economically feasible monitoring strategy in water bodies at risk.

We designed a set of ML-based regression soft-sensors to infer Chl-*a* fluorescence by disentangling the intrinsic relationships between the four mentioned low-cost input variables (i.e. water temperature, pH, EC and system battery). The ML models used in the soft-sensors were trained and tested with the collected three-year data and their performance was studied during different months of a year to analyze the varying environmental situation of each month. We hypothesized that these Chl-*a* soft-sensors could be applied as online-predictor of Chl-*a* or as a backup sensor to be used when the physical Chl-*a* sensor is malfunctioning. Furthermore, we built a component capable of triggering level 1 alerts on HABs outbreaks. This alarm system was created to signal when the predicted value is greater than 10 $\upmu$g/L, which is equivalent to the level 1 alarm according to the decision tree provided by the WHO.

As a novel approach with respect to existing works, we propose to implement the soft-sensor with a set of ML algorithms presenting moderate levels of complexity in order to analyze their performance with respect to their computational needs in terms of memory and CPU. It is worth noting that CPU and memory efficient models tend to be more energy efficient, which is an essential factor in soft-sensors that will be powered by limited-capacity batteries. In addition, we created input and output aggregations in the selected ML models to investigate the increase in the inference performance they produced. Firstly, instead of predicting an instant sample of Chl-*a* that might contain a non-negligible amount of noise, our regressors were trained to predict statistical values (e.g., mean, median) of Chl-*a* as these statistics should be less likely to contain noise. These values were computed over periods of hours to study the generalization effects of output aggregation on the performance of the regression model. Secondly, new input features were generated by computing some statistics of the original low-cost variables using their values in previous hours. After adding these new features to the models, we investigated whether the new ML models could discover the possible temporal relationships contained in each input variable and whether the inclusion of the derived variables in the models could increase their performance.

The rest of the paper is structured as follows: “Machine learning models for soft-sensors” briefly describes the ML models used in our study. The methodology we followed during data acquisition and preprocessing and for the training and testing of ML models is detailed in “Method”. The results obtained in our experiments are depicted in “Results” followed by the discussion of main results in “Discussion” and conclusions in “Conclusions”.

## Machine learning models for soft-sensors

Data-driven soft-sensors can be implemented using different artificial intelligence techniques such as rigid rule-based expert systems, flexible ML models and complex deep learning architectures. The performance of a rule-based system is based on expert knowledge that is difficult to obtain and update and therefore, its intelligence is virtually fixed. On the contrary, ML systems are more flexible as they can extract features and their relationships using input variables, and map them to the desired output using statistical techniques. To extract the features to be used by ML models that best represent the underlying problem, a hand-crafted process that applies domain expert knowledge along with feature engineering heuristics must often be used. In order to avoid the limitation that supposes the ability of the expert to identify good features, complex representation learning approaches as deep learning techniques have been proposed to automatically learn the implicit useful representations or features from raw data without expert assistance. Although deep learning techniques usually obtain better results than traditional ML models^61,62^, they tend to consume more energy during their inference work and, in addition, larger and more expensive hardware infrastructures are required (e.g., additional GPUs to accelerate inferences). These limitations preclude their deployment in scenarios such as the one in our study, where (a) limited capacity batteries are available on the buoys and (b) the space and hardware required are also limited, as the buoys are intended as low-cost buoys to allow an scalable deployment of them in many different locations in the reservoir^30^. Hence, we selected a set of popular supervised ML regressors with different levels of complexity to evaluate their performance as data-driven soft-sensors.

To infer high-cost variables from low-cost variables, supervised ML regression models require for training and testing labeled datasets with a sufficient number of examples composed of the low-cost input variables and a high-cost variable to be inferred. To this purpose, a big dataset was used containing a high-cost (i.e. Chl-*a*) variable to be inferred and several low-cost variables (i.e. pH, water temperature, EC and system battery) used as input variables. Three well-known regression models with different degrees of complexity, and therefore, different computational needs in terms of memory and CPU, were trained and evaluated to infer the Chl-*a* variable: (i) Linear regression: a simple and interpretable model selected to contrast with the other nonlinear models; (ii) Classification and Regression Trees (CART): a simple algorithm for building a decision tree selected as a representative of non-linear and interpretable models; and (iii) Random-Forest: a powerful, complex, non-linear and ensemble type model with some degree of interpretability selected as one of the most popular and efficient representatives of the state-of-the-art methods in classical ML.

Note that, although rare, a linear relationship between the input and output space should not be discarded in early stages of the analysis. In fact, over short periods of time, a certain level of linearity has been observed in these variables in^63^. For that reason, we did not discard linear models (Linear Regression) from our study for comparison with more complex models (CART and Random Forests) as they can provide an advantage in model simplicity and speed of execution, which could compensate for their use in low-cost buoys. Therefore, in our study, we included linear regression as representative of linear methods to compare them with other models and analyze the trade-off of using a simple and fast model versus its loss in accuracy and precision.

Linear Regression (LR)^64^ is a classical regression method borrowed from the statistics field by ML that attempts to model the relationship between a set of dependent and independent variables by fitting a linear equation to the observed data. The independent variables are considered to be explanatory variables. From an algorithmic standpoint, the goal of the training process is to find a linear combination of input (independent) variables that best predicts the output (dependent) variable (i.e. the inferred variable). In our study the dependent variable is Chl-*a* and the independent are pH, temperature, conductivity and system battery. It is worth noting that the model works better when every value of the independent variable is highly correlated with the dependent variables. Furthermore, adding L2-norm regularisation improves the generalisation ability of the model to datasets with outliers^65^. The model is computationally fast and frugal but assumes linearity between the dependent and independent variables, which may not be the case in many scenarios.

The Classification And Regression Tree (CART)^66^ model builds a decision tree and is capable of predicting continuous and discrete variables using both discrete and continuous input variables. CART learns and builds a binary tree of rules (if-else type) from the input variables of the dataset by splitting each node into two child nodes repeatedly. For regression predictive modeling problems the cost function that is minimized to choose split points is the sum squared error across all training samples that fall within a leaf. The recursive binary splitting procedure stops when a minimum count on the number of training instances is assigned to each leaf node. Predictions are made by traversing the binary tree given a new input record and landing in one of the leafs. The output variable is computed by averaging the values of the leaf. CART is considered an interpretable model due to its rule-tree type construction that allows to understand why each prediction is made. In addition, it is possible to use the model to evaluate the importance of the input variables. When predicting, the CART model is slower than a linear regression model that only needs to compute the dot product of two vectors. In the case of CART models, the inference process implies to traverse the tree in a dichotomic search evaluating the boolean conditions in each node. The advantage of this method is that it can find nonlinear relationships between input and output variables.

The Random-Forest (RF) model^67^ is a supervised learning algorithm that uses an ensemble learning method. The RF ensemble model is composed of a set of decision tree models that are considered as submodels. The good performance of RF is based on the fact that a large number of relatively uncorrelated models (decision trees) operating as a committee outperform any of the individual constituent models. The reason for this effect is that the trees protect each other from their individual errors as long as they do not constantly all make errors in the same direction. Current RF implementation uses CART models as submodels. To reduce the correlation between the submodels, the RF model is trained using the bagging technique which consists in selecting a random subset of variables for each submodel. The RF model predicts by averaging the results of the submodels and is more robust and generalises better with outliers than the CART model. Although the inference process of a RF model involves computing an inference for each of its component trees, this process can be straightforwardly parallelised on a regular multi-core CPU by assigning the prediction of each tree to a different core.

On top of these regression models we built a simple alarm system to detect whether the inferred output level of Chl-*a* was higher than 10 $\upmu$g/L, as the alarm level 1 proposed by the WHO^13^. It should be noted that, based on what is proposed by WHO, a vigilance level and a level 2 alert could be also built when Chl-*a* exceeds $1 \upmu \hbox {g}$/L and 50 $\upmu$g/L, respectively. In the case of the vigilance level, we did not elaborate a warning system because in most of the sampling period the Chl-*a* exceeded $1\,\upmu \hbox {g}$/L. On the other hand, we did not implement an alert level 2 because of the poor performance of the selected ML models obtained in the validation process. The reason for this poor performance has to do with the fact that these values were rare in our experiments and, in general, infrequent data are not well predicted by classical ML models as they are mainly based on statistical learning principles.

## Method

In this section we detail the method we followed in our study. First, we describe the buoy location and data acquisition processes, whereby data were collected from two points on a freshwater reservoir every 15 min over a 3-year period. Then, we detail the data cleaning and preprocessing we applied to the collected data. Within the preprocessing phase, we detail two innovative contributions: (i) the input and output aggregation we designed to boost the performance of ML models and (ii) a complex data set splitting procedure we designed to make better use of the available data. After that, we depict which ML models were selected for the study, how ML models were trained and tested jointly with the metrics used for measuring the peformance of inferences and level 1 alarm signaling. Furthermore, we describe what hyperparameters were selected for each ML model and how they were tuned and validated.

### Buoy location and data acquisition

**Figure 1:**
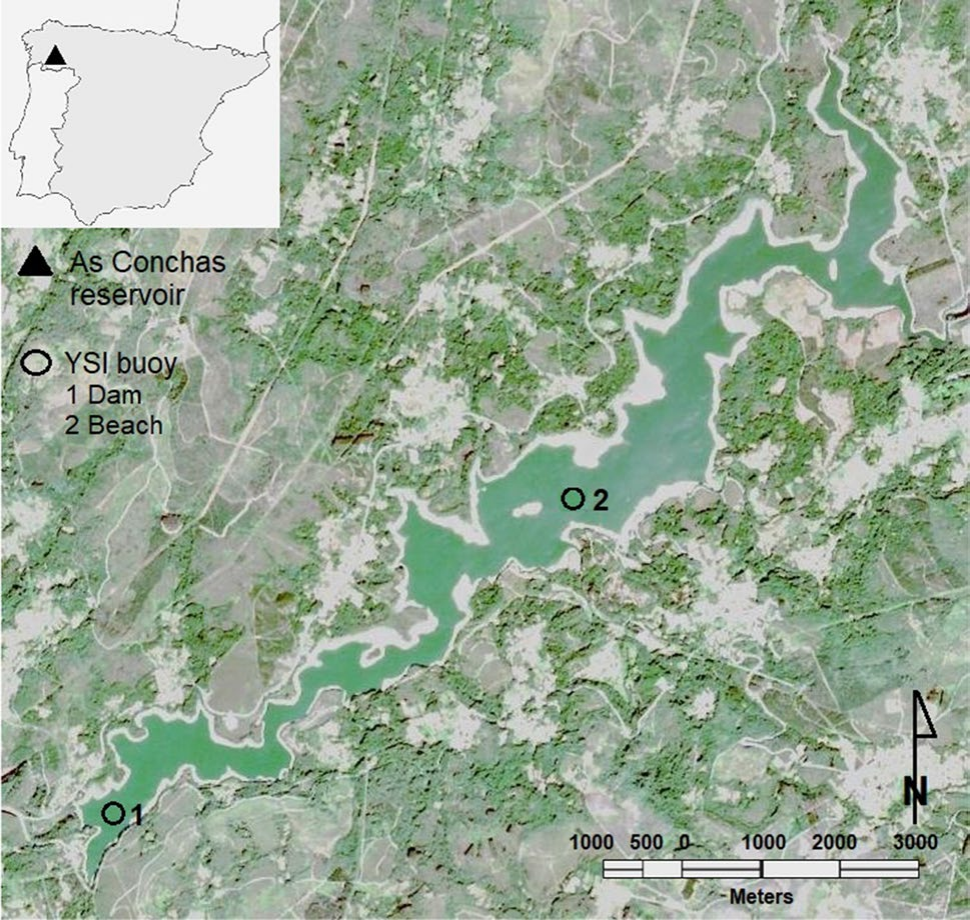
Buoys location in As Conchas reservoir. (1) Dam buoy (41$^{\circ }$56’41.78”N; 8$^{\circ }$1’47.96”W) and (2) beach buoy (41$^{\circ }$57’56.57”N; 7$^{\circ }$59’15.27”W). The image was taken by sentinel 2 on September 25, 2018, a cloudless day. The daily mean Chl-*a* collected by the beach buoy on that day was 24.70 $\upmu$g/L, while at the dam buoy it was 41.98 $\upmu$g/L. The satellite image was obtained from the ESA (European Space Agency) Sentinel database using the Application Platform (SNAP) that is an open access software developed by Brockmann Consult, SkyWatch and C-S under the Creative Commons CC BY-SA 3.0 IGO license (https://step.esa.int/main/download/snap-download/, https://www.esa.int/ESA_Multimedia/Copyright_Notice_Images). The image was generated in RGB, channels B4, B3 and B2 (pixel resolution 10m). Water colors were highlighted using the histogram (RGB) and normalized. Finally, the study area was cropped and this figure was composed.

The As Conchas reservoir is an eutrophic freshwater body located the “Baixa Limia-Serra do Xurés” Natural Park, belonging to the Miño-Sil River Basin District in Galicia, NW Spain. It has a retention capacity of 80 H$m^3$ of water and its depth varies considerably between the shore and the center of the reservoir, reaching depths of up to 32 m at times of maximum retention. According to the basin organization in charge of reservoir management (Miño-Sil Hydrographic Confederation), secchi disk depth generally ranged from 2 to 6 m between May and October in the three years of the study. Two commercial EM1250 buoys (Xylem Analytics) were anchored in two fixed locations in the center of the reservoir (Fig. 1), named beach buoy (41$^{\circ }$57’56.57”N; 7$^{\circ }$59’15.27”W) and dam buoy (41$^{\circ }$56’41.78”N; 8$^{\circ }$ 1’47.96”W), whose separation distance was approximately 4 km. Each buoy was equipped with a YSI multiparametric probe (EXO3) containing an automatic brush and sensors for measuring Chl-*a* (total algae fluorescence sensor), pH, water temperature and EC (for more detailed information about the sensors see Morón-López et al.^30^). The installed sensors and probes were regularly maintained according to the manufacturer’s recommendations for cleaning, calibration and replacement rates. The use of solar panels allowed the system to be completely autonomous, and the battery power was used as an indicator of daylight hours. The multiparametric probes were placed at approximately 1m of depth (standard depth for EM1250 buoy) and the datalogger was set up to collect data every 15 min.

### Data preprocessing

Two hundred thousand records were collected from the two buoys over three years, at intervals of 15 min. Before being used to train the ML models, the collected data were preprocessed in the following pipeline: (i) the data were cleaned and missing data were removed; (ii) a subset of features were selected and augmented with a statistical time-related aggregation of independent (input) and dependent (output) variables; (iii) the resultant dataset was normalized to accelerate model learning during the training phase; and (iv) the normalized dataset was divided into three datasets (training, testing and validation) using a complex procedure to facilitate further analysis at monthly granularity.

#### Data cleaning

Data were curated by correcting and eliminating measurement errors. It was observed that some variables had values outside their limits. First, values that exceeded up to 5% of the limits of the variable were replaced by their nearest boundary, as the error seems to be caused by some calibration problem in the physical sensor. Second, the samples in which a value exceeded more than 5% or was absent were removed, as the measure was clearly incorrect and some severe problem could have appeared on the sensor during the measurement. After applying the cleaning process, approximately 5% of the original dataset was removed.

#### Feature selection and augmentation

Five features were selected from the cleaned dataset: pH, EC, water temperature, system battery and Chl-*a*. A statistical description of these features is presented in Table 1. The first four were used by ML models as input (independent) variables and the fifth (i.e., Chl-*a*) as output (dependent) variable to be inferred by the models. New features were generated by deriving time-related information from the input variables to make them more robust to outliers. Each original feature was aggregated over 1 hour and 24 hours, by computing five typical descriptive statistics: mean, standard deviation, median, minimum and maximum. Therefore, 4 input combinations were obtained: (i) *input_orig* (4 features): original features, (ii) *input_hour* (24 features): the original features and 1-hour aggregated statistics per feature, (iii) *input_day* (24 features): the original features and 24-h aggregated statistics per feature and (iv) *input_mix* (44 features): the original features and 1-h and 24-h aggregated statistics per feature. In addition, we derived a new set of output variables from the variable Chl-*a* by calculating its mean and median values to filter the noise that could be present in the instantaneous values of this variable. We analyzed whether the use of these statistics produced any beneficial effect on the performance of the soft-sensors or not. Then, four output variables were added to the original Chl-*a* value: (i) *output_orig*: Original Chl-*a* variable, (ii) *output_hour_mean*: Mean value of Chl-*a* aggregated for 1 h, (iii) *output_hour_median*: Median value of Chl-*a* aggregated for 1 hour, (iv) *output_day_mean*: Mean value of Chl-*a* aggregated for 1 day, (v) *output_day_median*: Median value of Chl-*a* aggregated for 1 day. The augmented input features and output features configurations were combined, generating 20 possible variations of the preprocessed dataset.

#### Data normalization

As the LR model performs better when all input variables follow a normal distribution, the dataset variables were normalized using the power transform^68^ function (to ensure that all variables follow the normal distribution) and a standard scaler (to ensure that all variables are in the same interval). We did not apply a typical normalization as the power transform has been reported to deal better with outlier values. Note that normalization was not needed for models based on decision trees, but regarding that this process does not penalise them during the training, we kept it for the three models to homogenize the training process.

#### Dataset splitting

Since the evolution of the studied biological systems depends on temporal trends (e.g., seasons of the year), we implemented a complex system of dataset separation in training, validation and testing to obtain a fine-grained evaluation of the three ML model families and thus be able to see their performance in the predictions made in different months. In the first instance, we discarded using a random split, as it is typically done in ML processes because the examples in the dataset are ordered in time and it is recommended in the literature not to use this random partitioning, as it could generate a considerable overfitting. Using the data from the first two years for training and validation, and thus leaving the last year for testing, would have been a good approximation, but we would only have one example from each month of the third year to compare the performance of each ML model family.

**Figure 2:**
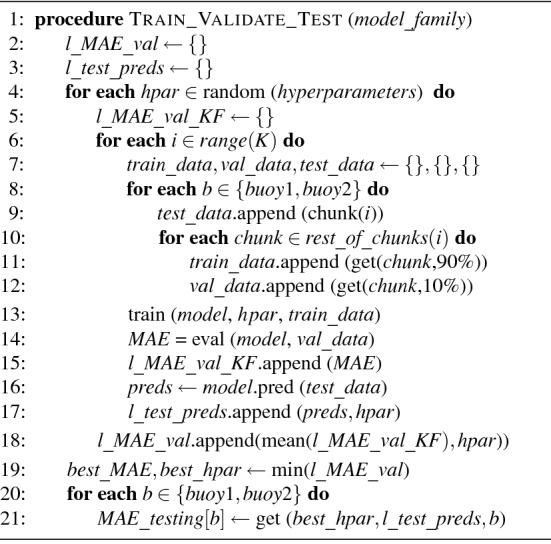
Dataset division.

Because of that, we decided to go a step further and see the performance of each model family in the three available occurrences of each month of the year, instead of just one occurrence in the third year. For this purpose, we designed a complex procedure (Fig. 2) inspired by the K-fold cross validation method but modified to maintain the temporal relationship of examples in the resulting datasets. Since the splitting method is integrated with the training and validation steps, we discuss the proposed algorithm in some detail in “Machine learning training and testing”.

**Table 1:** Statistical description of the collected data: number of collected data points (count), mean value, standard deviation value (std), minimum value (min), 25%, 50% and 75% percentile values, and maximum value (max).

	Temperature	EC	pH	System battery	Chl-*a*
Count	217133	217133	217133	217133	217133
Mean	15.10	67.26	7.59	13.31	8.98
Std	6.01	12.84	0.92	0.43	8.28
Min	5.37	34.69	4.41	12.74	0
25%	9.39	61.01	6.99	12.98	3.75
50%	14.25	69	7.29	13.14	6.63
75%	21.06	74.67	7.9	13.58	11.47
Max	28.56	99.52	10.31	14.84	135.84

Variables (columns): temperature ($^\circ$C), EC ($\mu$S/cm), pH (und), system battery (V) and Chl-*a* ($\upmu$g/L).

### Machine learning training and testing

The three ML model families (i.e., LR, CART and RF) were tuned using the Random Search heuristic^69^, which iteratively trains and evaluates models with randomly selected configurations of hyperparameters. Each family of ML models used a different set of hyperparameters: LR applied the L2 regularization coefficient between 0.0001 and 1.0; CART tuned the maximum depth of the decision tree between 1 and 500 without any pruning; and RF adjusted the maximum depth of the sub-models (*i.e.*, CART decision trees) using the same range as in CART models and the number of sub-models (estimators) between 2 and 200. The regression models were evaluated using the mean absolute error (MAE $= \frac{1}{n}\sum _{i=1}^{n} | y_{i}-{\hat{y}}_{i}|$, where ${\hat{y}}$ and *y* are the predicted and the expected values respectively). MAE maintains the scale and dimensions of the output variable, and therefore, it has a straightforward interpretation. After being tuned, the hyper-parameter configuration of each model that obtained the best results on a validation dataset was evaluated and analysed with a separate testing dataset to analyse the inference performance of all models when faced to unseen data.

Aiming to test ML models at a monthly granularity and to maximize the number of months to be used during the testing phase, we designed a novel process inspired in the K-fold cross validation process to separate the training, validation and testing datasets. Typical data splitting as described in the literature would use the first two years of data for training and validation and only the months of the third year would be used for testing purposes. In contrast, our algorithm (Fig. 2) splits the original dataset in a different way to test the performance of a model in a particular month using the three months available in the 3-year dataset. Lines 4 to 19 describe the validation process done to train a model and select the best combination of hyperparameters for a family of models (i.e., LR, CART or RF), and lines 20 and 21 describes the testing process done separately for each buoy. The validation process uses a novel process inspired in the K-fold cross validation method. The original dataset, which is not shuffled to preserve the temporal relationship of the examples, is divided into *K* chunks, one of which is used for testing and the rest for training and validation. Validation and training data are obtained at chunk level to preserve the temporal relationship of examples. For each K-fold iteration (lines 6 to 17) a new model is trained and validated. Therefore, a hyperparameter combination is represented by a set of *K* models trained each with a different combination of $K-1$ chunks, and its performance is the mean value of the performance of the *K* models. In this way, each combination of hyperparameters is tested against the three available months of the three-year dataset. Note that the best performing combination of hyperparameters is tested separately with each buoy (lines 20 and 21) to see the generalization capabilities of a model trained with these hyperparameters when used for inferring Chl-*a* in different buoys.

Despite not being a ML model *stricto sensu*, the alarm system built on top of the regression models can be analysed using typical classifier metrics as its output is a binary red/green flag signaling whether the inference of Chl-*a* has reached level 1 alarm or not. Therefore, we calculated the following metrics to quantify the performance of the alarm detector: *F*1-score, precision, recall and confusion matrix. *F*1-score is computed as the harmonic mean of precision and recall and can used as an aggregated metric to select the best performing regression model for the alarm system. Precision and recall as basic metrics can help interpret the false positive and negative ratios produced by a soft-sensor.

$${\text{Precision}}= \frac{\text {TP}}{\text {TP}+\text {FP}},\quad{\text{Recall}}= \frac{\text {TP}}{\text {TP}+\text {FN}}, \quad F1{\text{-score}}= \frac{2\cdot \text {Precision}\cdot \text {Recall}}{\text {Precision} + \text {Recall}},$$

$\text {TP}$ is the True Positives, FP is the False Positives, FN is the False Negatives

For training and testing all ML models, one off-the-shelf computer was used with 16 GB of RAM and an Intel(R) Core(TM) i5-9500 CPU running at 3.00 GHz. The experiments were coded using Python 3^70^ programming language and a set of commonly used scientific libraries: numpy 1.30.1^71^, pandas 1.2.4^72^ and scikit-learn 0.24.1^73^.

The collected dataset had 222, 000 records with 5 columns, and after cleaning and feature augmentation, the resulting dataset contained 5% fewer records and 51 columns (44 input features and 5 output features). We ran a complete round of experiments for each of the 20 combinations of inputs and outputs previously described in “Data preprocessing” to analyse the effects of the proposed data augmentation in the performance of ML models. In every round of experiments, 25 configurations of hyperparameters were randomly generated per ML model family and K-Fold procedure was configured with $K=10$. In total, 15, 000 ML models with 1500 unique hyperparameter settings were trained and validated. The results of all experiments are detailed in “Results”.

Table 2 summarizes the characterization of each ML model family with respect to the memory required to store the model, the time needed to train it and its inference speed. Results are segregated per input type, since the number of input features affects all three measured characteristics. As expected, it can be seen that complex models as RF are bigger in size and slower in inference time and training speed than simpler models as LR. Considering a deployment scenario similar to the one described in our study with space and hardware constrained buoys, the first two models (i.e., LR and CART) are small enough to be integrated in a reduced embedded system with sensors as they need less than 30 MB of RAM to be deployed. On the contrary, RF is much bigger requiring a significant amount of RAM memory to be stored ranging from 300 to 1000 MB. All training times are less than 1 minute which suggests that retraining processes to fix a data drift problem in a model can be done as soon as this problem is detected. However, deploying retrained models on remotely located buoys could pose logistical challenges. Although simpler models (such as LR) are 10 times faster than complex ones (such as RF) generating inferences, all models are fast enough to infer Chl-*a* at the expected 15 seconds granularity. Note that in the worst case, an RF model generates approximately 680, 000 inferences per second.

**Table 2:** Model size, Training time and Inference speed per ML model family and input set.

	Input features (num. features)	Model size (MB)		Training time (s)		Inference speed (million pred./s)	
		Mean	Max	Mean	Max	Mean	Min
Linear regression	*Input_orig.* (4)	0.000657	0.000658	0.05	0.180189	6.64	6.227645
	*Input_hour/day* (24)	0.000810	0.000811	1.64	3.120221	5.76	5.279960
	*Input_mix* (44)	0.000966	0.000966	3.25	5.379071	5.12	4.735821
CART	*Input_orig.* (4)	17.22	18.894751	0.53	0.549016	4.45	2.572063
	*Input_hour/day* (24)	16.63	18.920263	2.85	3.013313	4.08	2.377553
	*Input_mix* (44)	16.34	18.866186	5.26	5.626011	3.76	2.538655
Random-Forest	*input_orig.* (4)	355.28	2047.384612	4.39	11.311513	0.65	0.284171
	*Input_hour/day* (24)	354.10	2048.579925	24.76	61.137886	0.68	0.245368
	*Input_mix* (44)	347.99	2043.802215	45.83	113.191902	0.68	0.294159

## Results

In accordance with the objectives of this study, we conducted a set of experiments to evaluate: (i) a ML-based soft-sensor regressor capable of inferring the variable Chl-*a* from a set of easy to measure variables (i.e., pH, water temperature, EC and system battery) and (ii) a level 1 alarm system built on top of the soft-sensor. Three ML models were selected (i.e., LR, CART and RF) and several data aggregation techniques were explored. First, using only the four original variables as input to the models, and without doing any aggregation at the input or output, we compared the performance of the three ML-based regressors to a naïve model used as a baseline. Each month of the year was analysed separately to evaluate the influence of seasonal changes. Next, we evaluated the three ML models using as output two statistics (i.e., mean and median) of the output variable (i.e. Chl-*a*) to aggregate its values and dampen the stochastic variations that might appear in the physical measurement of Chl-*a*. Five statistics (i.e., maximum, minimum, median, mean and standard deviation) were then calculated for each input variable to add temporal information to the input of the ML models, and analyse whether or not this feature augmentation benefits the performance of the models. Finally, we analyzed the feature importance information provided by RF for all input variables, as this method revealed interesting non-linear relationships between input and output variables that initially could not be discovered using traditional linear correlation methods.

### ML models without aggregations

In this section, we shall explain the accuracy of the predictions obtained after training three ML models against the raw data collected, without further preprocessing. As a baseline, we selected a naïve model that predicts the Chl-*a* value as the mean of this value calculated using only the training data and never the testing data. It is worth noting that the baseline only has the utility of detecting very bad ML models and therefore being able to discard them. For that reason, we have selected the previously mentioned naïve model as a very conservative baseline. More complex baselines could be proposed in future work to have a more competitive ML model control method.

As commented in “Machine learning models for soft-sensors”, we used MAE and F1 score as quality metrics to analyze the performance of the ML models. The MAE metric relates to the regression problem and assesses whether the model is faithful to the overall set of Chl-*a* values. On the other hand, the F1-score classification metric measures the hits of the alarm signal from when the Chl-*a* value is greater than 10 $\mu$g/L (i.e. alarm level 1 according to the WHO). As can be seen from the results, both metrics did not always point to the same ML model as the best. When the model is used to trigger alarms, the F1-score metric should be taken into account, but if we are interested in being faithful to the original Chl-*a* values, the MAE metric should instead be considered.

**Figure 3:**
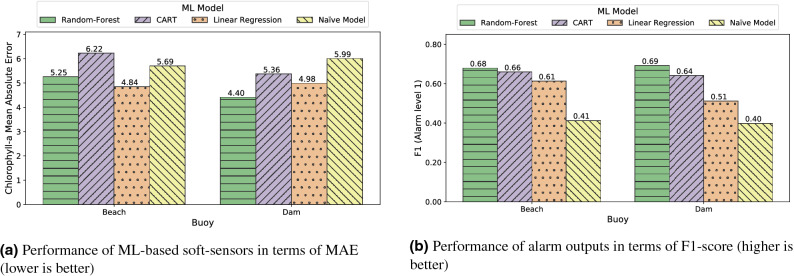
Comparison of the performance of ML models in Beach and Dam buoys. MAE calculated on ML-based soft sensors (**a**) and F1 calculated on alarm post-processing outputs (**b**).

**Figure 4:**
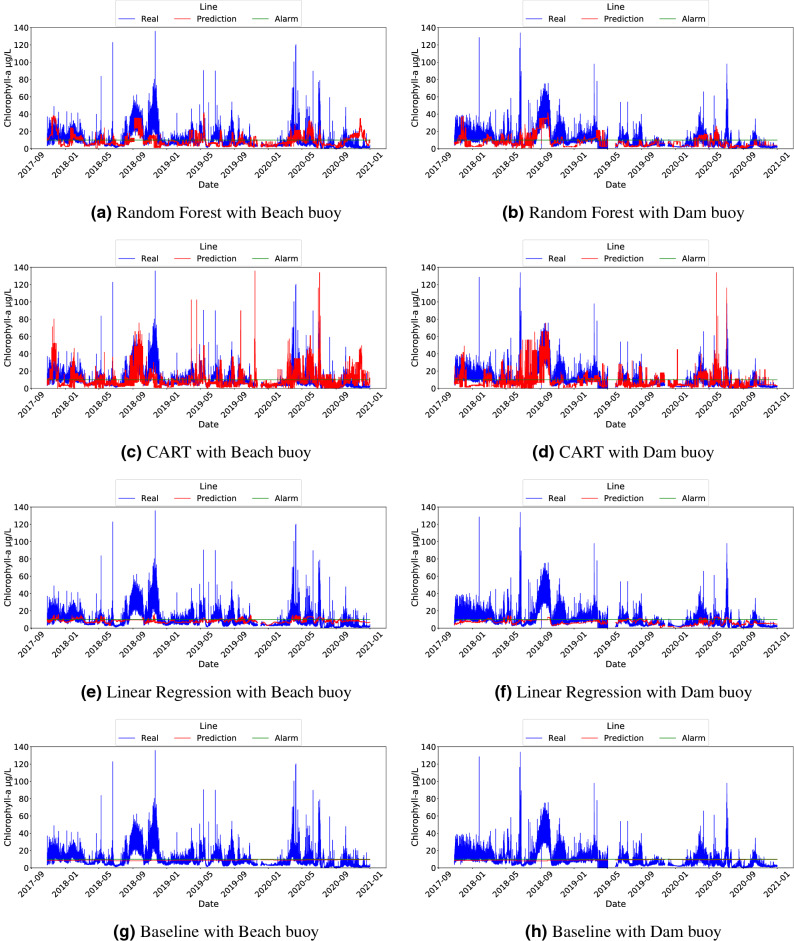
Line plots of Chl-*a* and ML predictions for each model and buoy (time is shown in x-axis and the Chl-*a* value in y-axis): (**a**) Random Forest with Beach buoy; (**b**) Random Forest with Dam buoy; (**c**) CART with Beach buoy; (**d**) CART with Dam buoy; (**e**) LR with Beach buoy; (**f**) LR with Dam buoy; (**g**) Baseline with Beach buoy; (**h**) Baseline with Dam buoy.

The results of the three models used, as well as the baseline, are shown in Figs. 3 and 4. It should be noted that Fig. 4 shows data using the x-axis to present the time dimension, which is a convenient way to detect the underlying trend and to identify fast variations of these trends in algae bloom scenarios. Although using scatter plots does not allow us to reflect the temporal behaviour of the data, the corresponding scatter plots for the three models and the baseline can be found in Fig. S2 in the Supplementary Material. In light of the results shown in Fig. 3, we observed that almost all models outperformed the baseline. From these data, we can conclude that RF was the best model for the task of triggering alarms and was also compelling when replicating the original sensor according to MAE. Another competitive model seems to be LR, which shows worse results in the classification task than RF, but outperformed it for the regression task in MAE. This discrepancy can be explained by means of the shape of Chl-*a*: the linear nature of LR models allows it to capture properly the mean of the output variable, leading to a good prediction on average. However, this linear behaviour prevents the LR model from adapting to the outliers that trigger the alarm (that is, probabilistically unexpected values greater than 10 $\upmu$g/L). Despite that, it is worth noting that LR is the lighter and less computationally expensive model. A separate mention deserves CART, which achieved even worse results than the baseline in the beach buoy in terms of MAE but compelling results in the classification task. The rationale behind this result is that the CART model is not very subtle when capturing the exact values of the output variable, but it correctly detects Chl-*a* outliers (due to fluorescence sensor interferences or erratic bloom changes). Despite that, CART has the advantage of being more interpretable than RF and requires fewer space resources.

To reinforce these conclusions, Fig. 4 shows the comparison between the inferences of each model and the Chl-*a* values measured by the fluorescence sensors, which visually corroborated the results of the MAE metrics in Fig. 3. The green horizontal line represents the Chl-*a* value at which the alarm is triggered (i.e. 10 $\upmu$g/L). This value was used to calculate the F1-score metric. In general, RF and CART models showed the best fit comparing to the original Chl-*a* variable, while both the LR and the Baseline models showed the worst. Notice that, although it seems in subfigure (c) that CART is better than RF in subfigure (a), there are many high values that do not coincide with the real values, as it is confirmed by the MAE metric in Fig. 3. Also, subfigures (e) and (f) shows how the LR model prediction did not reflect the actual sensor variability behaviour, in line with the aforementioned observation. It this sense, it is worth mentioning that in November 2018 and December 2020 noticeable discrepancies occurred at the beach buoy between the real and predicted Chl-*a* (Fig. 4). In November 2018, the fluorescence sensor detected Chl-*a* levels above 60–80 $\upmu$g/L several times, while the soft-sensor only exceeded 20 $\upmu$g/L using the CART model. In contrast, the Chl-*a* measured by the fluorescence sensor in December 2020 rarely exceeded 10 $\upmu$g/L, while the soft-sensor predicted values close to 40 $\upmu$g/L of Chl-*a* with RF and CART. In order to unravel the source of the error, we compared these results with the hand-collected data from the basin managers in the beach area. The Chl-*a* quantified in these samples was 43 and 11 $\upmu$g/L for November 2018 and December 2020, respectively (data shown in Figure S1 in the supplementary material). Therefore, our soft-sensor underestimated the Chl-*a* in November 2018, and overestimated it in December 2020. Nonetheless, it is important to notice that in both cases the soft-sensor triggered the alarm because the Chl-*a* exceeded 10 $\upmu$g/L.

#### Month based analysis

**Figure 5:**
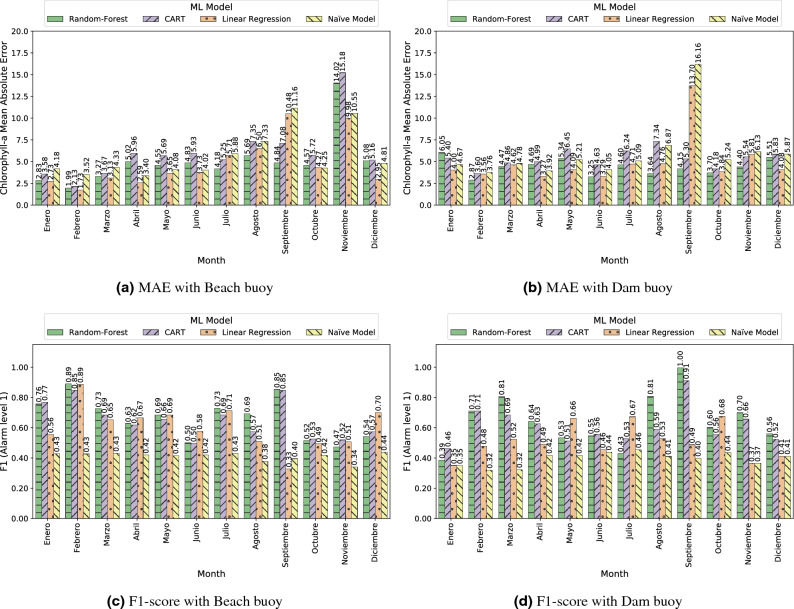
Comparison of ML models per month and per buoy: Beach and Dam buoys in terms of MAE (**a,b**); Beach and Dam buoys in terms of F1-score (**c,d**).

Figure 5 shows the comparison between MAE and F1 score for the tested models per month at each buoy. Considering that the months from June to September correspond to the warm season in the As Conchas reservoir, the models behaviour in those months is especially interesting because more intense blooms, and therefore, higher Chl-*a* values, are expected. During those months, the RF model showed relatively low MAE metrics and it provided good performances for the alarm signaling comparing to the other models. The F1-score was especially high in September, probably due to the constant exceeding of the 10 $\upmu$g/L threshold for Chl-*a* in that month. Indeed, between the end of August and the beginning of September blooms usually reach their maximum growth in this study, which is maintained until they decline in October. It is also remarkable that the F1-score was low in June and July at the beach and dam buoys, respectively. This could indicate that the rate of change of the input variables selected in this study is lower than that shown by Chl-*a*, and therefore, the ML model is less accurate at the onset of bloom. The RF model returned slightly better results than CART, whereas LR and the baseline models exhibited the worst results. Nonetheless, it is noteworthy that these models actually triggered the alarms more accurately than RF in July at the dam buoy. In fact, the RF model did not infer an increase of Chl-*a* above 10 µg/L during July 2020 in that location (see Fig. 4b), while the other models did. Similar situations occurred in June at the beach buoy, where LR predicted better than RF. This result highlights that there is not a single model that works perfectly for all cases. Therefore, this suggests that the combination of ML models according to bloom stage (i.e., onset, plateau and decay) should be considered to improve prediction performance. Because of the importance of reaching good inferences at the beginning of the bloom, future research in this direction will be served.

In the remaining months, particularly during the cold season between December to March, Chl-*a* values were usually lower, less variable and closer to the mean, so that the MAE and F1-score showed good outcomes, as observed for RF and CART models. It is also remarkable the high MAE metrics obtained for the beach buoy during November in all tested models, which is in line with the above-mentioned underestimation of the soft-sensor on that month in 2018. Likewise, the dam buoy exhibited high MAE metrics for LR and Baseline models in September. The fact that Chl-*a* was notably higher than the mean in September 2018 may explain these poor results. In summary, all ML models have proven to return better predictions than the baseline model in most cases, both for MAE and F1-score metrics.

### ML models with output aggregation

As variations of Chl-*a* values were observed in the order of hours, days, or even weeks, and the sampling protocol collected samples every 15 minutes, we applied some moving-window aggregation strategies to damp the stochastic variations and to extract the essential trend of the time series. For this purpose, we analyzed the effect of aggregating the output feature (i.e. Chl-*a*) through 1 hour and 24 hours periods using the mean and median as aggregation functions. Additionally, these aggregation procedures attempted to decrease the point errors of the fluorescence sensor, either due to errors in the electronics or misdetection of other Chl-*a*-containing artifacts (e.g. branches, leaves, unreal biomass, etc.). Concretely, by applying the median, we completely eliminated the effect of sample outliers, whereas the effect was only reduced when the mean was applied. In both cases, the loss of information after regularizing the time series was minimum.

**Figure 6:**
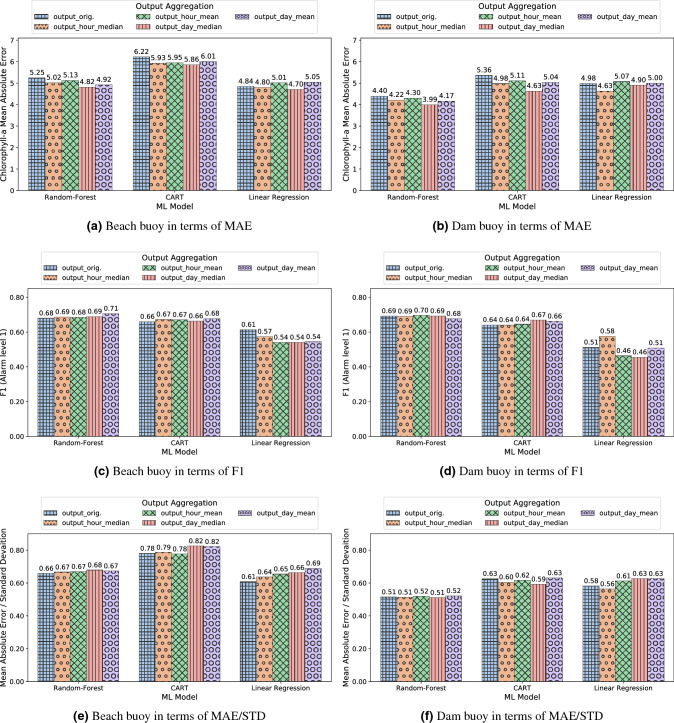
Comparison of output features aggregation with ML models: Beach buoy in terms of MAE (**a**) ); Dam buoy in terms of MAE (**b**); Beach buoy in terms of F1 (**c**); Dam buoy in terms of F1 (**d**); Beach buoy in terms of MAE/STD (**e**); Dam buoy in terms of MAE/STD (**f**).

**Figure 7:**
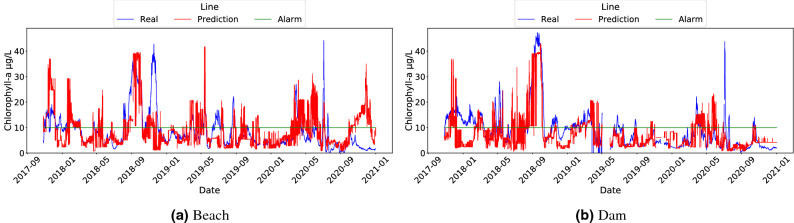
Line plots of Chl-*a* and RF predictions (time is shown in x-axis and the Chl-*a* value in y-axis) using the median of Chl-*a* computed over a day (output_day_median) as output for Beach (**a**) and Dam (**b**) buoys.

In light of the results plotted in Fig. 6, the comparison of the MAE and MAE / STD plots shows that, when aggregation to the Chl-*a* variable was applied, the results tended to exhibit a lower MAE. This could be due to the fact that they were less influenced by the Chl-*a* oscillations along the day, and therefore, the improvement of the MAE was proportional to the decrease of the standard deviation of the variable to be inferred. Specifically, in most cases the best results were observed when a median aggregation was applied within a 24-h period. These results showed the smallest variance in the output variable and provided the lowest MAE metrics. The results of Fig. 6 suggest that the best model was RF, since it maintained the best F1-score with a low MAE. CART performed worse than RF in terms of MAE and F1-score, but its F1-score values were better than those of LR. In the case of LR, this model showed an improvement of MAE with respect to CART in the beach buoy, but it exhibited the worst F1-scores in both buoys. These results are aligned with the fact that RF is a model with fewer prediction outliers than CART, as well as it gets the trends better than LR. Likewise, the output_day_median aggregation favored RF more, as it eliminated the outliers that significantly affected the evaluation of the original model without aggregation on the output variable. Fig. 7, using the x-axis to present the time dimension, shows a plot with the comparison of the Chl-*a* variable and RF inferences with output_day_median aggregation (the corresponding scatter plot can be found in the Fig. S3 in the Supplementary Material). It can be observed that, by using output aggregation, the behaviour of the Chl-*a* variable and its prediction are more similar than when no aggregation was performed (Fig. 4).

### ML models with input aggregation: features augmentation

**Figure 8:**
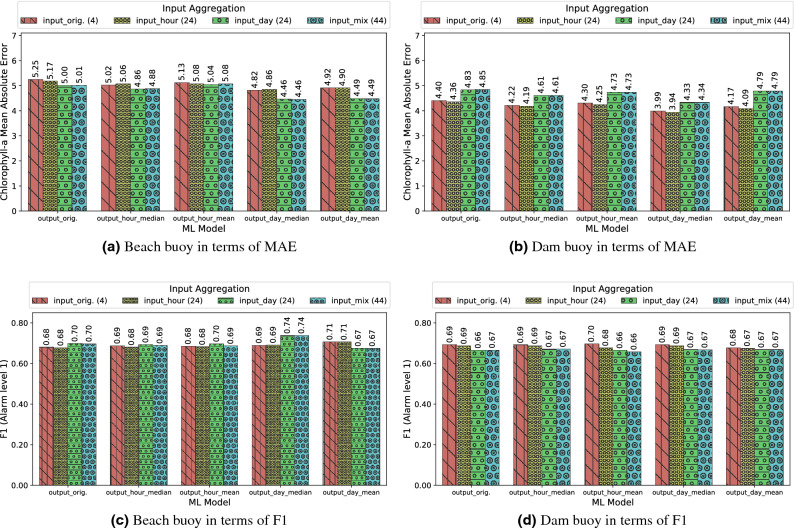
Comparison of various input aggregation and output aggregation methods using Random Forest: Beach buoy in terms of MAE (**a**); Dam buoy in terms of MAE (**b**); Beach buoy in terms of F1 (**c**); Dam buoy in terms of F1 (**d**).

**Figure 9:**
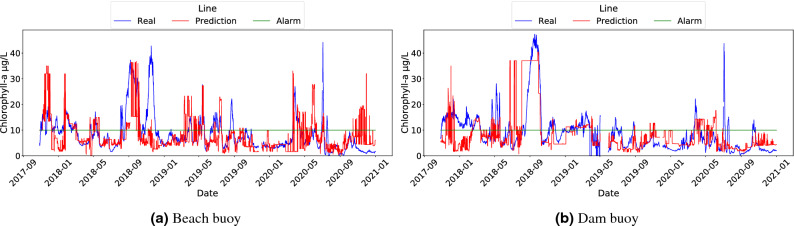
Line plots of Chl-*a* and RF predictions (time is shown in x-axis and the Chl-*a* value in y-axis) using the median of Chl-*a* over a day as the output variable (output_day_median) and aggregating all inputs over a day (input_day) for Beach buoy (**a**) and 1 h (input_hour) for Dam buoy (**b**).

**Figure 10:**
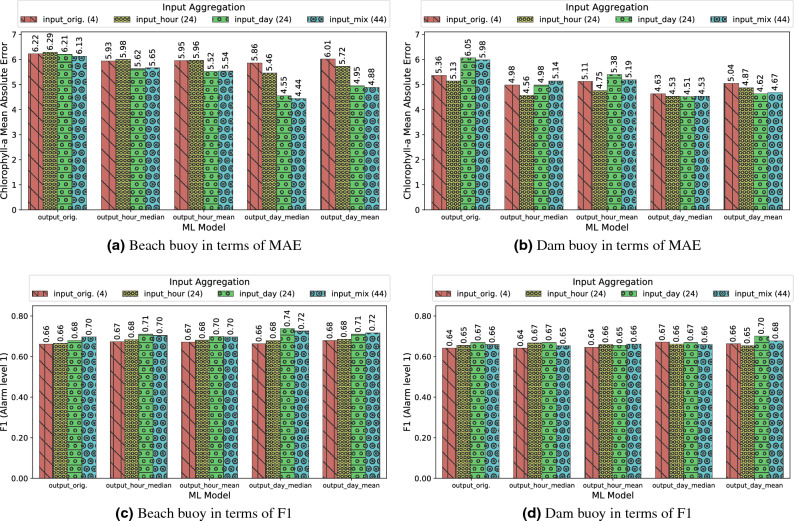
Comparison of various input aggregation and output aggregation methods using CART: Beach buoy in terms of MAE (**a**); Dam buoy in terms of MAE (**b**); Beach buoy in terms of F1 (**c**); Dam buoy in terms of F1 (**d**).

**Figure 11:**
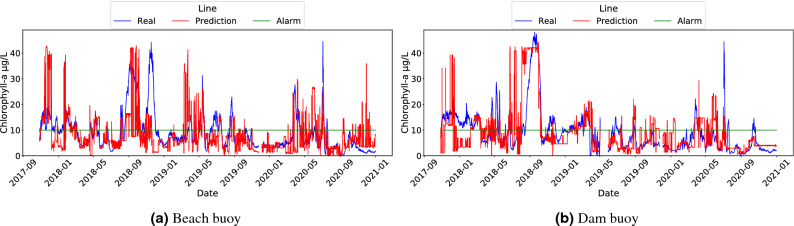
Line plots of Chl-*a* and CART predictions (time is shown in x-axis and the Chl-*a* value in y-axis), with the input aggregated over a day (input_day) for both buoys and using the median of Chl-*a* computed over a day (output_day_median) as output for Beach buoy (**a**) and the mean of Chl-*a* computed over a day (output_day_mean) for Dam buoy (**b**).

In some scenarios, it is known that conducting feature augmentation may improve the results of a ML task^74^. Feature augmentation is a procedure that consists of adding new features to the data that feed the ML model. Typically, these new features are obtained as a combination of the original ones, for example, through basic arithmetic operations. In the case of data with a strong temporal component, as the ones studied in this paper, it made sense to obtain these new features by summarizing the previous history of the time series. In particular, in this work we applied aggregations computing the mean, standard deviation, median, minimum, and maximum of each of the features of the time series over the last hour or the last 24 h. In the same vein, ML models were trained with the aggregate and non-aggregate output feature, as we did in “ML models with output aggregation” . The quality metric results of the RF and CART models applying different input and output aggregations are detailed in next subsections and presented in Figs. 8, 9, 10 and 11 .

#### Random forest (RF)

Figure 8 shows the comparison of the input and output aggregations for the RF model according to the regression and alarm tasks using MAE and F1-score metrics respectively. As can be seen in the figure, in the beach buoy the combination of input and output aggregations over a day improved both metrics, obtaining the best F1-score and the minimum MAE when the input was aggregated over a day (input_day) and the median of Chl-*a* was computed over a day(output_day_median). These aggregations achieved the reduction of several anomalous Chl-*a* peaks, leading to smaller errors during inferences, as can be seen in Fig. 9a. In contrast, the dam buoy did not show any improvement in the MAE and F1-score metrics after the input and output aggregations, and only a slightly lower MAE was achieved with one hour of input aggregation (input_hour) and the median of Chl-*a* computed over a day(output_day_median) as output. (the corresponding scatter plots for Fig. 9 scenarios are presented in Fig. S4 in the Supplementary Material). Future work should consider a separate analysis of errors by month to determine in which months and locations the model is generating the largest errors. These results suggest that the input aggregations in the dam buoy might over-emphasize past information, which implied a delay in triggering the alarms and led to a drop in F1-score. We speculate that the rationale behind this behaviour is that the dam area is deeper ($>10$ m) than the beach area, which means that the thermocline is more pronounced in the dam area. This thermocline prevented mixing and allowed a greater migratory range of bloom in the water column, which led to a greater variation of Chl-*a* throughout the day resulting in less reliable input aggregates. The vertical-profiling data provided by the basin managers supported this hypothesis. Furthermore, even though in the case of the dam buoy, the combination of the aggregated input and output data slightly worsened the results, the output_day_median aggregations accounted for a greater improvement in the beach buoy (roughly 5% of improvement). Therefore, these results suggest that the addition of aggregated information to the RF model could have more benefits than drawbacks.

#### Decision tree (CART)

The results of the CART model combining input and outputs aggregations are shown in Fig. 10. The beach buoy results show that, as with RF, the input_day and output_day_median aggregations significantly improved the MAE and F1-score metrics. Unlike RF, in the dam buoy the input_day and output_day_mean aggregation also improved both metrics. Overall, the aggregations made with the CART model are nearly equal than the best RF result at the beach buoy according to the F1-score, also improving the RF results for the MAE results (by tenths), but not the MAE metric at the dam buoy. These results point out that the complex RF model –a probabilistic model based on bagging– is much more robust in situations where the data are poorly treated (so it performs better without aggregation). However, when the data are well treated and filtered, a simpler ML model as CART could be more accurate due to the avoidance of overfitting.

Finally, Fig. 11 shows the line plots of the best CART results for each beach (i.e., input_day and output_day_median aggregations) and dam buoys (i.e., input_day and output_day_mean aggregations). In this figure, it can be observed that the CART model trained on an intensively processed dataset exhibited fewer noticeable errors, resulting in a performance close to that of RF models. The corresponding scatter plots are shown in Fig. S5 in Supplementary Material.

**Figure 12:**
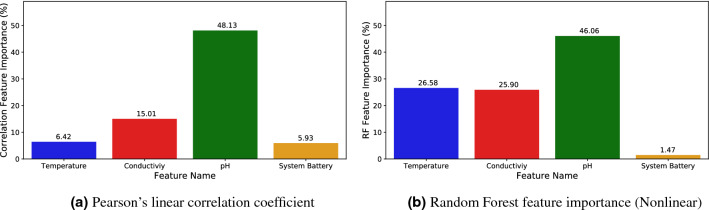
Feature importance of the original features for Chl-*a* prediction using linear (Pearson’s coefficient **a**) and nonlinear (Random Forest Feature importance **b**) calculations. Note that the percentage value of a feature can only be compared with respect to the other features in the same graph as each graph represents different coefficients.

**Figure 13:**
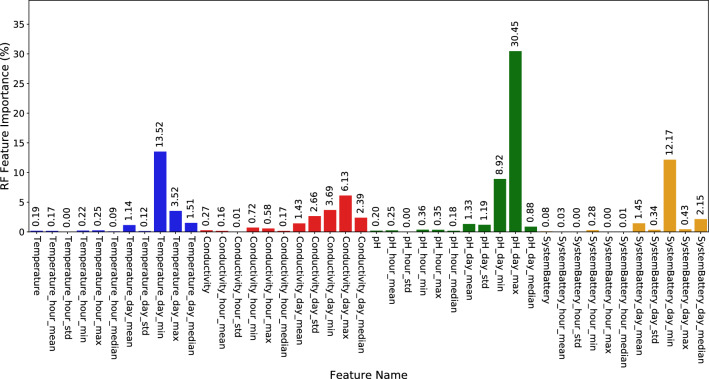
Feature importance generated by the best Random Forest model for Chl-*a* prediction using original and augmented input features.

### Feature importance analysis

Additionally, a study of the importance of the input features (i.e., water temperature, pH, EC, and system battery) for Chl-*a* inference was carried out using the values of their correlation with the collected Chl-*a* by the fluorescence sensor and the RF model weights. The results are shown in Fig. 12, where both Pearson’s linear correlation coefficient (subplot Fig. 12a) and feature importance as predicted by RF (subplot Fig. 12b) are analyzed. Recall that the correlation analysis is inherently a linear method, while the RF weights are obtained through a non-linear process. Indeed, the RF model made of a set of small models (classification trees) that predict by voting or averaging. Each small model ranks with a randomly selected subset of input features (for each small model). Roughly speaking, the more used a feature is to make a prediction, the more important. For this reason, this method of measuring the importance of features strongly depends on the good quality of the model on the assumption that the features are not highly correlated with each other. Input features that are highly correlated with each other have an equal importance value. In this case, we assumed that the trained RF model had good quality and that, among the four input features, only pH and water temperature presented a moderate correlation (0.68).

According to Fig. 12, the pH showed the greatest influence in the ML model. The algal and cyanobacterial growth cause alkaline pH through the photosynthetic metabolism^33^, and hence, this result illustrates the high capacity of these microorganisms to modify the pH of surface water. On the other hand, water temperature and EC had a very similar significance value according to RF, whereas the absolute value of the correlation between them was low (0.123) in the whole dataset. The effect of water temperature in algal and, in particular, cyanobacterial ecophysiology is well-known, since many of these microorganisms reach their maximum growth rate at temperatures between 25 and $35\,^\circ$C^9^. In addition, the hydrological changes that occurred in the reservoir during the three years of study are also related to HAB outbreaks and influenced the analysis of the features importance. For instance, decreases in EC may be related to rainfall and discharges from rivers flowing into the reservoir, while increases in EC in the warm season may be due to drought events. These changes cause mixing of the water column and subsequent stratification, both related to the release of nutrients from the sediment and their further accumulation in surface water, which promote HABs^75^. At the same time, an increase in the number of cells could also lead to an increase in EC, since many compounds released by microorganisms may conduct electricity. Lastly, the system battery had the least influence in the Chl-*a* inference by RF. This could be because the batteries were fully recharged during the first hours of daylight, and therefore, the model did not find a strong relationship between voltage increase and recorded Chl-*a* changes.

Feature augmentation was carried out by applying statistical methods (“Method”) to assess whether temporal information improves the results of ML models. Figure 13 shows the importance of the augmented features together with the original ones according to the RF non-linear model. This model was trained without applying aggregation to the output feature. It can be observed in the figure the clear influence of the new features, especially the minimum and maximum daily aggregation features. The pH variable appears to be the most important feature as before, being the maximum pH the most important one. As discussed above, the greater the number of photosynthetic microorganisms, the higher the pH and Chl-*a* values in the surface water. Therefore, the maximum value of pH is revealing information to the model. The aggregation of the minimum temperature also becomes more important, possibly because it provides seasonal and day/night period information, which correlates better with Chl-*a* levels. Low temperatures during the night or in cold seasons imply low biological activity and therefore, lower Chl-*a* values. In contrast, feature augmentation did not substantially improve the importance of EC variable, probably because there were no significant changes in their daily and hourly value. Interestingly, the system battery became more important by including the minimum system battery level than if we only considered the original features (see Fig. 12b). The level of battery discharge is related to the length of day and night, the time of year (i.e., in temperate zones, more daylight hours in summer and less in winter), as well as climatic interferences such as cloud cover. Thus, the lowest battery levels would be achieved on cloudy days, in winter (fewer daylight hours) and at night. Because the blooms, and hence Chl-*a* values, are also influenced by these factors, the inclusion of this feature boosted the importance of the system battery in the designed Chl-*a* soft-sensor. Given the mandatory inclusion of solar panels and batteries in any autonomous and AHFM system, this result highlights the great usefulness of this parameter for Chl-*a* soft-sensors based on ML models.

Finally, we conducted an additional experiment to analyze what happens to the RF model when the best aggregate features were selected. To this end, we made a selection of the best features based on Fig. 13 to train new RF models and compare them with the best results of the previous RF models. This comparison is shown in Table 3. Two sets of features 3-AGG and 5-AGG were selected. The 3-AGG set representing $56.14\%$ of total importance was composed of the three best features: pH_day_max, Temperature_day_min, and SystemBatery_day_min. Set 5-AGG representing $71.19\%$ of total importance included the next two best features in addition to those in selected in the 3-AGG set: Conductivity_max_day and pH_min_day. The 5-AGG set contained at least one feature of each type and included two pH features because the pH_day_min variable was of greater importance than any of the Conductivity features. The selected output feature was the median of Chl-*a* computed over a day (output_day_median) since the models using it had previously obtained the best results. In general, it can be seen that, based on the predictive capabilities of alarm level 1 using the F1 score, the new RF models using the two subsets (3-AGG and 5-AGG) did not outperform the original RF models, and only in the case of the beach buoy the results were similar to the non-aggregated input feature set. However, most of the MAE values of the RF models using the reduced feature subsets were slightly better than those of the original RF models. This improvement in MAE can be explained by the fact that these reduced feature subsets were composed of aggregated features that had fewer outliers. Therefore, if a better MAE value is preferable to a better F1-score value, it is preferable to employ a model that uses one of the reduced feature subsets. Note that some models have a higher MSE despite having a lower MAE than other models. This may be because they have made several large errors in some predictions, as the MSE strongly penalizes these high errors. Finally, it was observed that the precision and recall values (used to calculate the F1-score) generated more false negatives than false positives in the case of the Dam buoy, and more false positives than false negatives in the case of the Beach buoy.

**Table 3:** Summary table with the best Random Forest results obtained for various features configurations (input and output aggregations) and buoys.

Experiment configuration			Chlorophyll-*a*		Alarm level 1		
Input	Output	Buoy	MAE	MSE	F1	Presicion	Recall
NO-AGG	NO-AGG	BEACH	5.246	66.816	0.679	0.676	0.683
NO-AGG	Day-median	BEACH	4.816	53.652	0.689	0.685	0.694
ALL-AGG	Day-median	BEACH	4.456	50.293	0.737	0.759	0.724
3-AGG	Day-median	BEACH	4.415	46.227	0.679	0.677	0.680
5-AGG	Day-median	BEACH	4.366	43.971	0.669	0.671	0.668
NO-AGG	NO-AGG	DAM	4.403	42.519	0.692	0.729	0.681
NO-AGG	Day-median	DAM	3.993	33.384	0.692	0.734	0.680
ALL-AGG	Day-median	DAM	4.336	46.973	0.667	0.690	0.659
3-AGG	Day-median	DAM	3.962	42.286	0.668	0.732	0.658
5-AGG	Day-median	DAM	4.234	31.260	0.641	0.714	0.635

Mean absolute error (MAE) and mean squared error (MSE) are Chlorophyll-*a* regression metrics. F1-score, Precision and Recall are Alarm level 1 classification metrics.

## Discussion

Many studies have tried to predict HABs by collecting massive data through manual sampling^51,54,56^, AHFM systems^16,17,60^, and satellite imagery^76–78^. However, such data-driven models are useful for the water bodies for which their equation is derived but present large uncertainties when applied to different water bodies^79^. This work does not intend to develop a predictive model for a specific waterbody, but to propose the development of soft-sensors based on easy-to-measure variables that allow, after a period of data collection and model training, the inference of Chl-*a* and the generation of a warning system to support manual sampling. According to the WHO thresholds^13^, alert level 1 is reached when Chl-*a* is equal or higher than 10 µg/L and the presence of problematic cyanobacteria is confirmed by microscopic analyses. Then, to continue with risk assessment and management, eventually reaching alert level 2 (i.e., 50 µg/L of Chl-*a*), genetic and toxicity analyses are required. Given that there are currently no effective sensor options for assessing this type of analysis in the field remotely and in real time^18^, the use of large soft-sensors networks could be a cost-effective and efficient way to reduce the spatiotemporal uncertainties of manual sampling.

In sharp contrast with other proposals, our work proposed the design of ML-based regression soft-sensors to infer Chl-*a* using water temperature, pH, EC and system battery, as input low-cost water quality variables. On top of the soft-sensor, we designed a level 1 alarm generator. Since the soft-sensors should be integrated in buoys with constrained resources and with limited battery capacity, instead of choosing complex deep learning algorithms that will require a significant amount of energy, space and computational resources, we selected a set of simpler ML algorithms, such as LR, CART and RF, that present a good balance between inference performance and complexity.

The proposed soft-sensors were tuned using a new algorithm that we designed inspired by the *K*-fold cross validation procedure but that maintains the temporal relationship of the data. Models were trained and tested using data collected from two buoys located in different locations (beach and dam) in the reservoir, which revealed different model performances of the same model when tested separately in each buoy. Because the dam area is deeper than the beach area ($> 10$ m), we speculate that the greater vertical migration of blooms in the dam area generated greater variations in Chl-*a* that were more difficult to predict accurately. Further research should be conducted to provide ML models with sufficient generalization capabilities in order to perform similarly in such different conditions. More data or data grouped in time series could be beneficial for training more complex models that can extract this information from the input variables and perform in a similar way independently of the location of the buoy.

Using only the original variables (water temperature, pH, EC and system battery) as input to the ML models, we conclude that RF is the best performing model for both tasks, inferring the Chl-*a* variable and triggering the level 1 alarm. Both LR and the Baseline (a naïve approach returning the observed mean value of Chl-*a*) obtained decent results inferring the value of this variable as they simply predicted its normal behaviour. It is worth noting that RF was successful in predicting many of the changes that appeared in the variable, and on the contrary, LR and the baseline model failed to infer any change of it. CART performance was not good in either of both tasks, although the model was able to detect changes in the values of the inferred variable. However, due to a lack of generalization of this model, the overall figures for MAE and *F*1-score were even worse than those obtained by LR.

During the cold season (from December to March) the values of Chl-*a* were lower, less variable and closer to the mean, which resulted in good performance of the models and, in particular, that of RF. On the other hand, a detailed analysis of the months with the most intense blooms (from June to September) reinforces the better performance of RF over the rest of models when inferring the value of Chl-*a*. However, LR was better signaling the level 1 alarm in July, which suggests that there is no single model that performs better in all months and in the two tasks. Future work could investigate combining several models in an ensemble to cope with this problem. In view of these results, and if only the original input variables can be used, it seems convenient to deploy RF for good inference performance despite its slight computational overhead compared to LR and CART.

To enhance the performance of the selected models we designed input and output aggregation methods. Assuming that Chl-*a* values should not experience fast variations in the range of seconds, and aiming to damp the stochastic variations that can appear in the physical measurements (e.g., failures in the electronics or misdetection of artifacts such as branches or leaves), we designed a moving-window aggregation strategy in which the output variable was replaced with the median and the mean value of the original output variable (Chl-*a*) for one hour or one day. In general, all three models benefit from this aggregation in terms of robustness and generalization, since outliers of Chl-*a* are filtered out and the models do not have to learn them. Consistent with previous results, RF was also the best performing model in both tasks (inference and alarm triggering) when output aggregation was applied.

Regarding that the data of our study has a strong temporal component, we derived new input features containing such temporal information. We computed five statistics for each of the original variables (i.e., mean, standard deviation, median, minimum and maximum) for the last hour and day to input this past information into the models. With input aggregation, CART results in both tasks (MAE and F1-score) improved notably in both buoys. However, RF results only improved in the beach buoy. We conjecture that in the dam buoy the augmented variables overemphasized the model towards them, which downplayed the importance of the original variables. In contrast, since the original beach data were more noisy and with more outliers, adding the augmented features helped RF to generalize and improve its results in this scenario. It is worth noting that a complex probabilistic model as RF is much more robust when data is not preprocessed, and therefore, if the input data is reasonably clean and does not suffer from errors or outliers, it performs better without the proposed input aggregation. Future work should investigate how to add this kind of augmented data without affecting in any circumstance the performance of the RF model.

We analized the relationships between input and output variables using the feature importance information provided by RF models at the end of their training. It is worth noting that the feature importance provided by an RF model provides revealing information about nonlinear relationships between the input variables and the inferred variable that complement the linear relationships obtained by typical correlation analyses (e.g., calculating Pearson’s linear correlation coefficient). Using only the original variables, the obtained RF feature importance results were coincident with previous linear correlation analyses in that pH is strongly correlated with the inferred variable (Chl-*a*). However, RF feature importance revealed existing nonlinear relationships between water temperature and EC with Chl-*a* that did not appear so vividly in the linear correlation analysis. In “Feature importance analysis” we provide a biological explanation of these nonlinear relationships that were revealed and quantified only when RF feature importance analysis was done. In addition, feature importance analysis using augmented input variables was applied to the models, which revealed non-obvious relationships with their corresponding biological explanations. Consistently, pH also appeared as the most important (correlated) variable with Chl-*a*. However, the aggregated minimum values of the water temperature and system battery appeared as variables strongly correlated with Chl-*a*. We conjecture that it is highly likely that these variables provide some kind of seasonal and day/night period information that could be exploited by RF to obtain more accurate Chl-*a* inferences. In the light of these results, we concluded that the inclusion of these two aggregated variables boosted the performance of RF when inferring Chl-*a*.

In the “Introduction” we discussed recent proposals for applying ML regression models to infer Chl-*a* in oceans, rivers, lakes and reservoirs. Among them, it is worth noting that Barzegar’s et al. work^60^ is close to our study, but we clearly differentiate from it in the following aspects:

1. We implemented on top of the soft-sensors a level 1 alarm system for Chl-*a* values according to WHO recommendations.
2. They used a single point for data collection and our study used two sampling points through the buoys placed at different locations in the reservoir. This is important for assessing the ability of the models to infer Chl-*a* throughout the waterbody, where changes in hydrologic variables may play a role in the recorded and inferred Chl-a.
3. They used only one-year data, which prevented to do a reliable validation on how the model will behave along next years, as they used data from the same months both for training and testing the ML models. In contrast, we collected data for three years, which allowed us to analise in detail the seasonality aspects of the Chl-*a* values, since for each month we collected samples at three different years.
4. As an interesting novelty, we used input and output data aggregations which produced an observable increase in the ML performance. Several input variables contained relevant temporal relationships between the inferred variable and them, but these relationships were not present in the isolated samples collected at a particular instant (e.g., seasonal and day/night periods of the temperature and the battery status). Only when we augmented the input variables with a set of complementary statistics for each input variable (maximum, minimum, median, mean and standard deviation) did these temporal relationships appear and could be exploited by the ML models to increase their performance. In addition, the aggregation of the output variable also produced beneficial effects on ML performance. We applied moving-window aggregation strategies (calculating the mean and the median in periods of 1 and 24 hours) to damp the stochastic variations and to extract the essential trend of the time series of Chl-*a*, which definitely helped to better train the ML models. These aggregated values decreased the point errors of the physical Chl-*a* fluorescence sensor and in the case of the median, completely eliminated the detrimental effect of outliers.
5. They applied complex deep learning models (a combination of CNN and LSTM networks) but we used simpler ML models to be less demanding in terms of hardware resources and energy consumed. Both, hardware resources and energy consumption, are crucial when soft sensors are going to be deployed on small buoys with limited-capacity batteries. In our study we demonstrated that a complex and energy-demanding deep learning model is not necessary to obtain an adequate performance, as we achieved decent performance using simpler ML models. In addition, we have shown that real-time inferences can be obtained using simpler ML models that need only modest hardware infrastructures to be deployed. For example, RF models can be parallelised straightforwardly using the available cores of an off-the-shelf Intel/AMD CPU, but on the contrary, deep learning models need additional and not cheap GPU cards to be run efficiently with some degree of parallelization.

## Conclusions

It is important to note that the physical sensors tested in this study are commonplace due to their low cost, low maintenance, easy calibration, and wide availability in the water monitoring device market. In addition, many manufacturers have developed dual devices that quantify water temperature and EC in a single sensor. This makes it possible to use, for example, multiparameter probes with a smaller number of ports, which means a lower price, or other low-cost customizable alternatives available on the market^30^. Therefore, the development of Chl-*a* soft-sensors based on these basic variables is an attractive option to design a long-term monitoring strategy, where price and technical knowledge for sensor handling are not an obstacle.

In our work we demonstrated that it is feasible to design a low-cost and energy-efficient soft-sensor to infer Chl-*a*, in conjunction with a 1 level alarm system for HABs warning, using a supervised ML regressors that uses as input a small number of variables monitored with low cost physical sensors. In particular, RF regressors achieved a good balance between inference performance and a frugal use of resources (i.e., battery consumption, hardware and physical space). It is worth noting that more complex models such as the ones based on deep learning techniques could obtain a better performance inferring Chl-*a* and predicting the level 1 alarm, but the power consumption and specialized hardware required would preclude their use in the resource-constrained scenarios such as those we are considering in our study.

In light of the promising results obtained in our study, interesting applications for a Chl-*a* soft-sensor can be devised. Firstly, when it is desired to increase the number of sampling points, the deployment of cheap Chl-*a* soft-sensors could be an alternative to expensive fluorescence sensors for Chl-*a*. For example, a low-cost monitoring strategy could be to use a single Chl-*a* sensor to train the ML models at a specific location, and then deploy the cheap soft-sensors in place, and relocate the Chl-*a* sensor to another location for further training of other soft-sensors. Secondly, the proposed Chl-*a* soft-sensor could be useful for assessing underperformance of deployed fluorescence sensors, either due to sensor malfunction (i.e., as a safety backup) or underestimation of actual Chl-*a* concentration when blooms accumulate in areas other than where the fluorescence sensors are placed (accumulation at shores or with deeper Chl-*a* maximum). It is worth noting that it is also possible for cheap sensors to malfunction, but since it would be economically feasible to increase their number (increased redundancy), they could democratically identify the malfunctioning soft-sensors to be replaced and filter their incorrect values applying a typical voting strategy.

Considering that the warm season is the most important for Chl-*a* inference, as this is when the greatest increase in Chl-*a* could be expected because bloom-forming algae and cyanobacteria are more active, the performance of the RF model was remarkable. During this season, basin management agencies must increase the frequency of manual sampling, hence our study demonstrates that soft-sensor networks would be accurate during periods of intense HABs, which could help reduce the efforts required to conduct such intensive manual sampling. On the other hand, the level 1 alarm system build on top of the Chl-*a* soft-sensor may also be of great interest for the implementation of smart warning systems in economically depressed areas affected by HABs. Furthermore, the alarm component can help to implement an alert smart system to make manual sampling more effective, indicating when it is necessary to collect samples and avoiding going to the study area at times when there are no blooms or it is not interesting to go. This, in turn, would decrease the cost of analysis, as samples would only be collected and analyzed when necessary. It should be noted that manual sampling cannot be totally replaced either by our soft-sensors or by current more expensive monitoring alternatives.

In addition to the previous discussions, this manuscript points to several interesting challenges to be investigated in future works:

- This work proposed a level 1 alarm system according to the decision tree provided by the WHO, but a level 2 alarm system capable of predicting Chl-*a* values greater than 50 µg/L would be also beneficial. This second level requires ML regressors capable of accurately inferring large and sudden changes of Chl-*a*. Therefore, more complex ML architectures (e.g., ensemble models) should be investigated to increase the performance of the ML regressors, while maintaining moderate resource usage (power consumption, and hardware and space requirements).
- In our study we collected data from three years in order to train and test the ML models with a sufficient number of examples. The question is whether it is necessary to collect three years of data, or perhaps half the time would achieve similar results. In other words, with the collected data, what is the number of data for which the addition of more data does not significantly change the accuracy of the model? Being able to characterize this parameter is important considering the significant effort usually required for massive environmental data collection. In addition, due to the high price of fluorescence sensors for Chl-*a*, knowing the minimum collecting time to obtain good estimates would allow the reuse of fluorescence sensors to train other water bodies in the shorter term. Unfortunately, the dynamics of the variables studied in our work are so complex and variable in each scenario that there is no conclusive answer to this question. With respect to the optimal size of the data to be used during training and validation in ML tasks, it is commonly accepted in the literature that the more the better and in particular, deep learning models clearly outperform traditional ML models when the training data is in the big data regime (i.e., hundreds of thousands of examples). Furthermore, adding more data than necessary to the training process is usually not harmful since ML learning is statistical in nature and therefore extracts the essence of the statistical behavior of the data. However, future work should explore the application of transfer learning techniques to incrementally add data to a model previously trained with a limited data set. In this way, a model initially trained with a small amount of data could be incrementally enriched with additional data without the need for full training as soon as more data were collected.
- In normal situations, we can assume that seasonal patterns will not suffer relevant degradation in the short and medium term, which could change the behaviour of HABs and thus alter the accuracy of model inferences. Therefore, ML models are not expected to require retraining in short periods of time (e.g., days or weeks), and only after the occurrence of a substantial change in the environment would be necessary to retrain the models. Nonetheless, future work should investigate the application of well-established techniques (e.g., Kolmogorov-Smirnov test and Kullback-Liebler divergences) to automatically detect these data drift problems and trigger the process of updating obsolete ML models.
- It should be noted that if the necessary data are available, the retraining time of the ML models used in our study does not exceed 2 minutes (Table 2) in a lab using off-the-shelf hardware. However, provisioning the retrained ML models on a large number of buoys located in hard-to-reach locations can involve considerable effort and cost. In this context, it would be interesting to investigate the application of recently appeared low-power IoT communication protocols for wireless sensors (e.g.. LoRaWAN) to enable an automatic provisioning method for remote updating of ML models on such hard-to-reach buoys.

## Supplementary Information

Supplementary Information.

## Data Availability

The data of this work have been collected from *As Conchas* reservoir which is an eutrophic freshwater body located in *Baixa Limia-Serra do Xurés* Natural Park, belonging to the Miño-Sil River Basin District in Galicia, NW Spain (for more detailed information see “Buoy location and data acquisition” ). The code and data used to develop the experiments in this work can be found in the following repository: https://github.com/stanislavvakaruk/Chlorophyll_soft-sensor_machine_learning_models.
