# Supplementary material for: Chlorophyll soft-sensor based on machine learning models for algal bloom predictions
Source: Sci Rep. 2022 Aug 8;12:13529. doi: 10.1038/s41598-022-17299-5 (PMC9360045; doi:10.1038/s41598-022-17299-5)
Supplement: Supplementary file 1 — Supplementary Information. [file 41598_2022_17299_MOESM1_ESM.pdf]

Supplementary material

Chlorophyll soft-sensor based on machine learning models for algal bloom predictions

Alberto **Mozo**[a], Jesús **Morón-López**[b], Stanislav **Vakaruk**[a], Ángel G. **Pompa-Pernía**[c], Angel **González-Prieto**[d], Juan Antonio **Pascual Aguilar**[c], Sandra **Gómez-Canaval**[a] and Juan Manuel **Ortiz**[c]

[a]*Universidad Politécnica de Madrid, Spain*
[b]*European Regional Centre for Ecohydrology of the Polish Academy of Sciences, Poland*
[c]*IMDEA Water Institute, Spain*
[d]*Universidad Complutense de Madrid, Spain*

A.1 Manual sampling

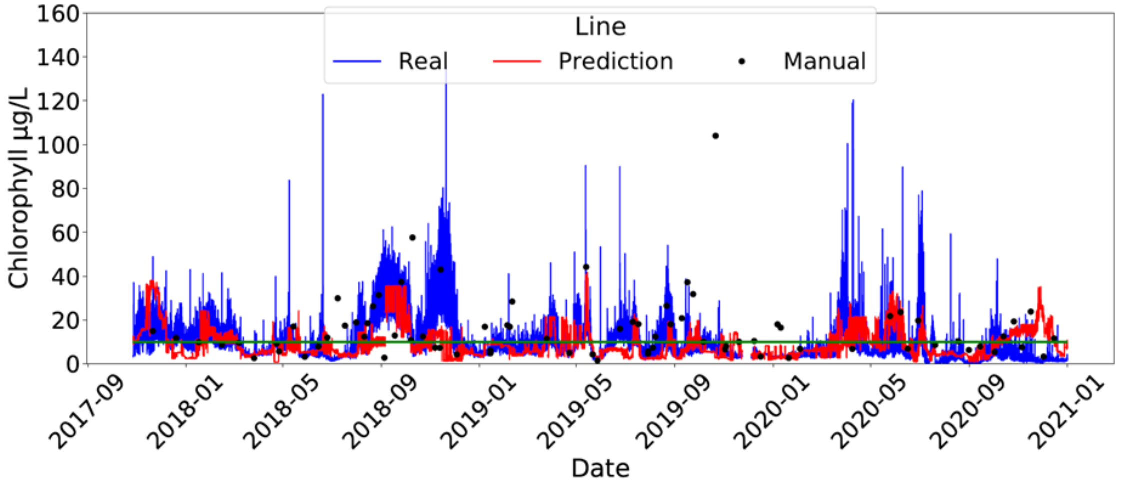

(a) Beach area

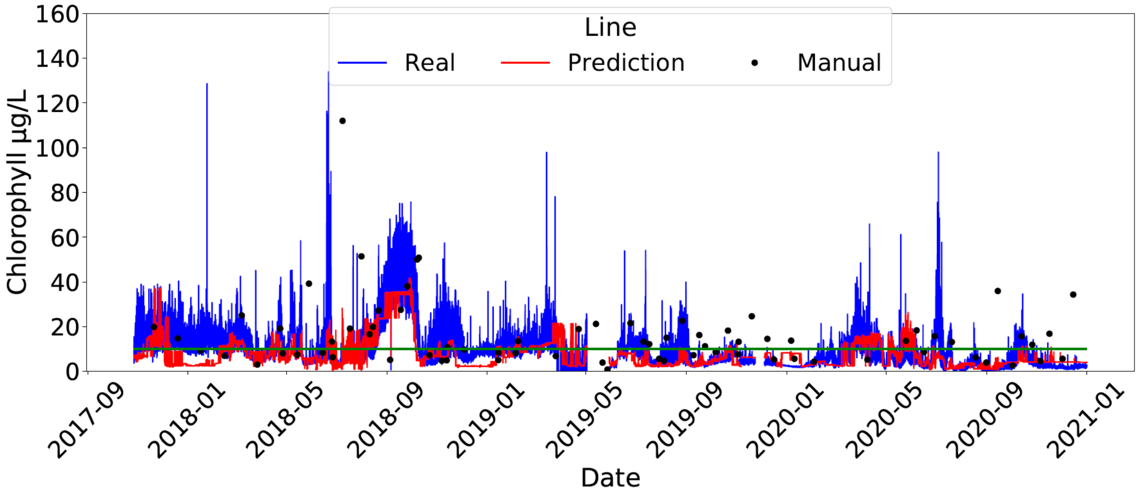

(b) Dam area

Figure S1: Plots of Chl-*a* (time is shown in x-axis and the Chl-a value in y-axis) measured by manual sampling by the basin organization in charge of reservoir management (black dots), physical fluorescence sensors (blue lines) and the soft-sensor based on the Random Forest model (red lines) for the beach (a) and dam area (b). It is important to note that, although the measurements were taken in the same area, the manual sampling was carried out on the shore, while the buoys were placed in the central area of the beach and the dam.

A.2 Scatter Plots

For the sake of completeness, in this appendix we include the scatterplots of the obtained predictions for each of the tested models in the four scenarios analyzed in this work. However, it is worth pointing out that these types of representations are not well-suited for the problem under study for the following reasons:

- The scatterplots cannot represent the time dependence of the data. The analyzed dataset is not a raw random sample of points, but a sample of points of a time series. For this reason, more important than predicting the right value is detecting the underlying trend and identifying the fast variations of these trends in algae bloom scenarios. This temporal behaviour is not reflected in these scatterplots.

- The scatterplots do not show any information about the density of the results. Since we are dealing with thousands of samples that are stacked at the same point, it is not possible to track how many values are around the diagonal nor their density variation.

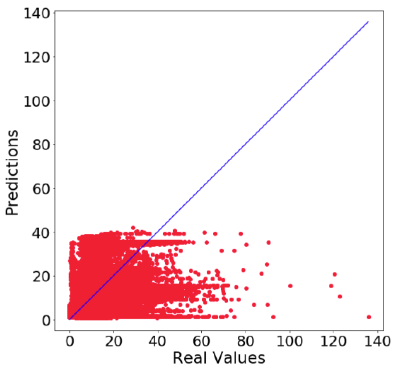

(a) Random Forest with Beach buoy

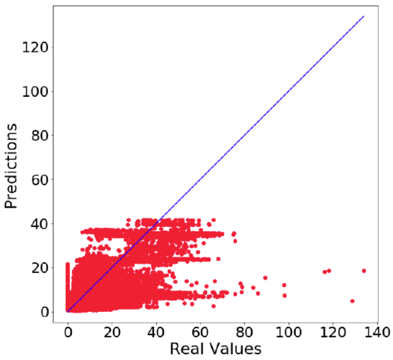

(b) Random Forest with Dam buoy

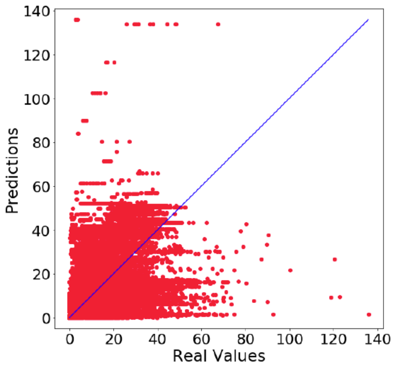

(c) CART with Beach buoy

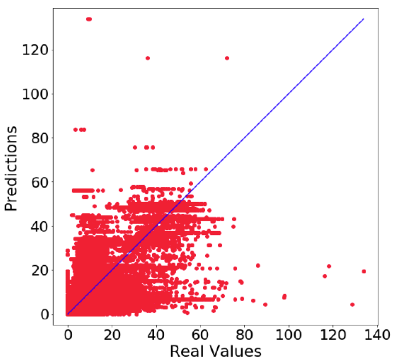

(d) CART with Dam buoy

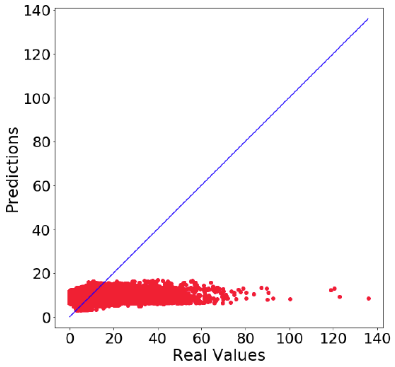

(e) Linear Regression with Beach buoy

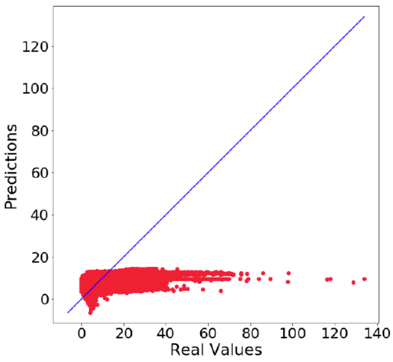

(f) Linear Regression with Dam buoy

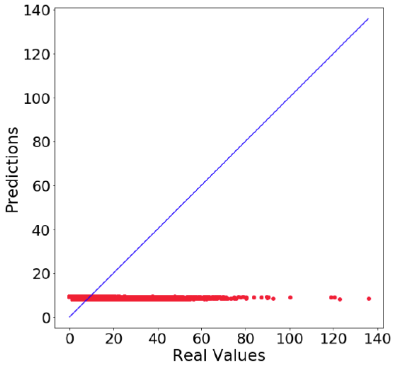

(g) Baseline with Beach buoy

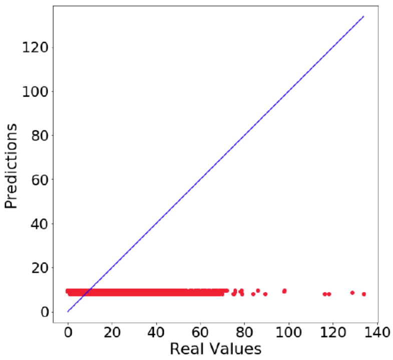

(h) Baseline with Dam buoy

Figure S2: Scatter plots of each ML model and each buoy: (a) Random Forest with Beach buoy; (b) Random Forest with Dam buoy; (c) CART with Beach buoy; (d) CART with Dam buoy; (e) LR with Beach buoy; (f) LR with Dam buoy; (g) Baseline with Beach buoy; (h) Baseline with Dam buoy.

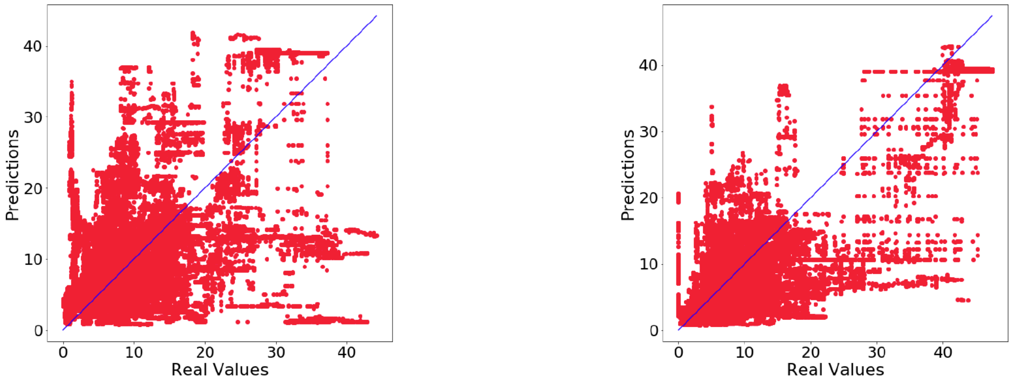

(a) Beach **(b)** Dam

Figure S3: Scatter plot of Chl-a (x-axis) and RF prediction (y-axis) with output_day_median aggregation for Beach (a) and Dam (b) buoys

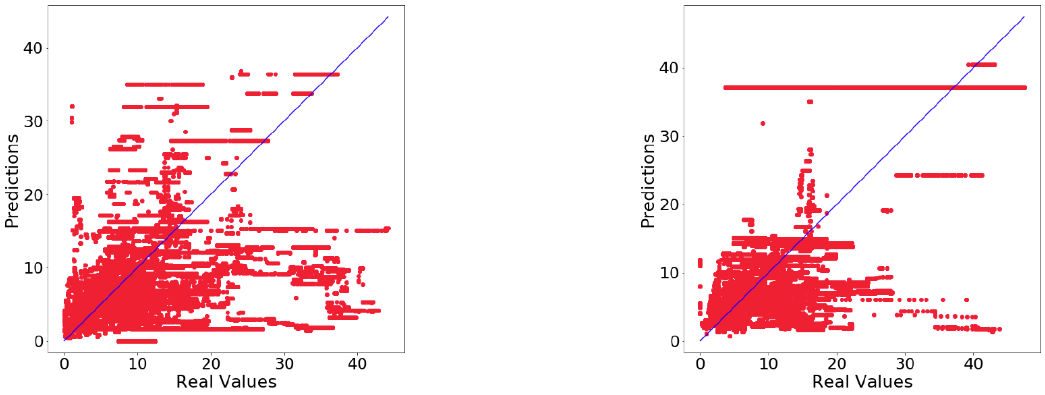

(a) Beach buoy **(b)** Dam buoy

Figure S4: Scatter plots of the input_day aggregated by applying median Chl-a and all aggregations input with RF model for beach buoy (a) and dam buoy (b}

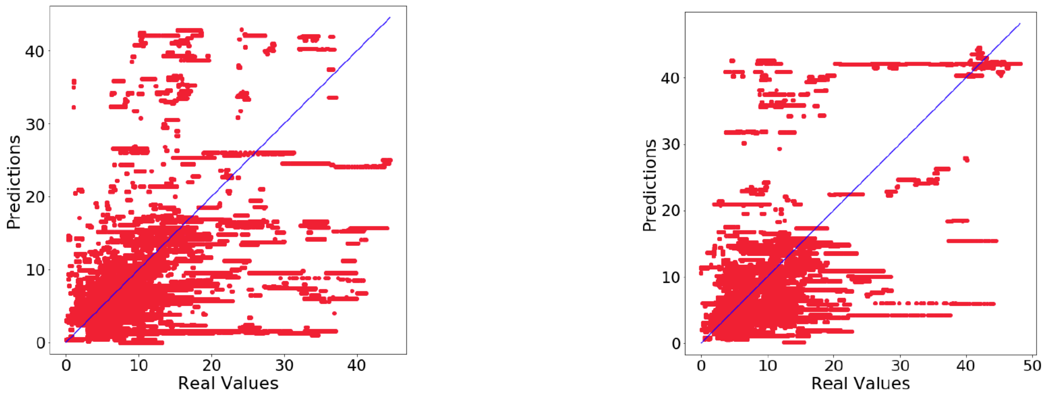

(a) Beach buoy **(b)** Dam buoy

Figure S5: Scatter plot of Chl-a (x-axis) and CART prediction (y-axis), with input_mix and output_day_median aggregations for Beach buoy (a) and output_day_mean aggregation for Dam buoy (b).
